# Use of pain drawing as an assessment tool of sciatica for patients with single level lumbar disc herniation

**DOI:** 10.1186/s40064-016-2981-z

**Published:** 2016-08-09

**Authors:** Toshiya Tachibana, Keishi Maruo, Shinichi Inoue, Fumihiro Arizumi, Kazuki Kusuyama, Shinichi Yoshiya

**Affiliations:** Department of Orthopaedic Surgery, Hyogo College of Medicine, 1-1 Mukogawa-cho, Nishinomiya, Hyogo 663-8501 Japan

**Keywords:** Pain drawing, Sciatica, Lumbar disc herniation, Conservative treatment

## Abstract

The objectives of this study were to examine the diagnostic accuracy of pain drawing (PD) in determining the level of involvement and to investigate how the quantitative evaluation results of PD using a grid score (GS) correlates with the results of other clinical evaluation measures in diagnosis and assessment of patients with lumber disc herniation (LDH) involving a single nerve root. Thirty-one patients with single level LDH who were diagnosed and conservatively treated by the first author constituted the study population. In order to assess the diagnostic accuracy of PD, the level of involvement as determined by PD was compared to the final diagnosis. In 26 of the 31 patients who could be followed for more than 6 months of conservative treatment, the GS in PD evaluation was compared to the score assessed by the Japanese Orthopaedic Association scoring system for low back pain (JOA score) and the visual analog scale (VAS) both before and after the treatment. The overall diagnostic accuracy of PD for the determination of the affected level averaged 68.8 %, and the accuracy was higher at the L4/5 and L5/S levels than the L2/3 and L3/4 levels. The average values of VAS and GS significantly decreased and the JOA score significantly improved after the treatment. Moreover, a significant correlation was demonstrated between the scores derived from these three evaluation measures. The present study indicated the potential usefulness of PD in clinical assessment during the treatment course.

## Background

In our clinical experiences, pain drawing (PD) has been effectively utilized to detect the affected level because the area of pain indicated in PD corresponds to the distribution of the affected nerve root. Moreover, patients’ perception of symptom improvement during the treatment course has also been assessed with PD. To date, there have been a number of studies examining the significance of PD in the diagnosis and evaluation of patients with various low back disorders. However, these previous reports mostly examined the efficacy of PD in psychological assessments (Dzioba and Doxey 1984; Gatchel et al. 1986; McNeill et al. 1986; Ohlund et al. 1996; Ohnmeiss et al. 1996; Ransford et al. 1976; Taylor et al. 1984; Uden et al. 1988), and there have been no studies that have specifically analyzed the efficacy of the use of PD in the clinical evaluation of patients with sciatica. Therefore, in this study, we analyzed the significance of PD in the diagnosis and assessment of patients with sciatica. In order to examine the patient population of uniform pathology, only patients with confirmed diagnosis of single root lumbar disc herniation (LDH) were included in the study. The first objective of this study was to examine whether the level of the involved nerve roots could be diagnosed using PD in this patient population. The second objective was to determine how the quantitative assessment of PD using a grid score (GS) correlates with the results of other clinical evaluation measures such as validated clinical score and visual analog scale (VAS) treatments.

## Methods

**Fig. 1:**
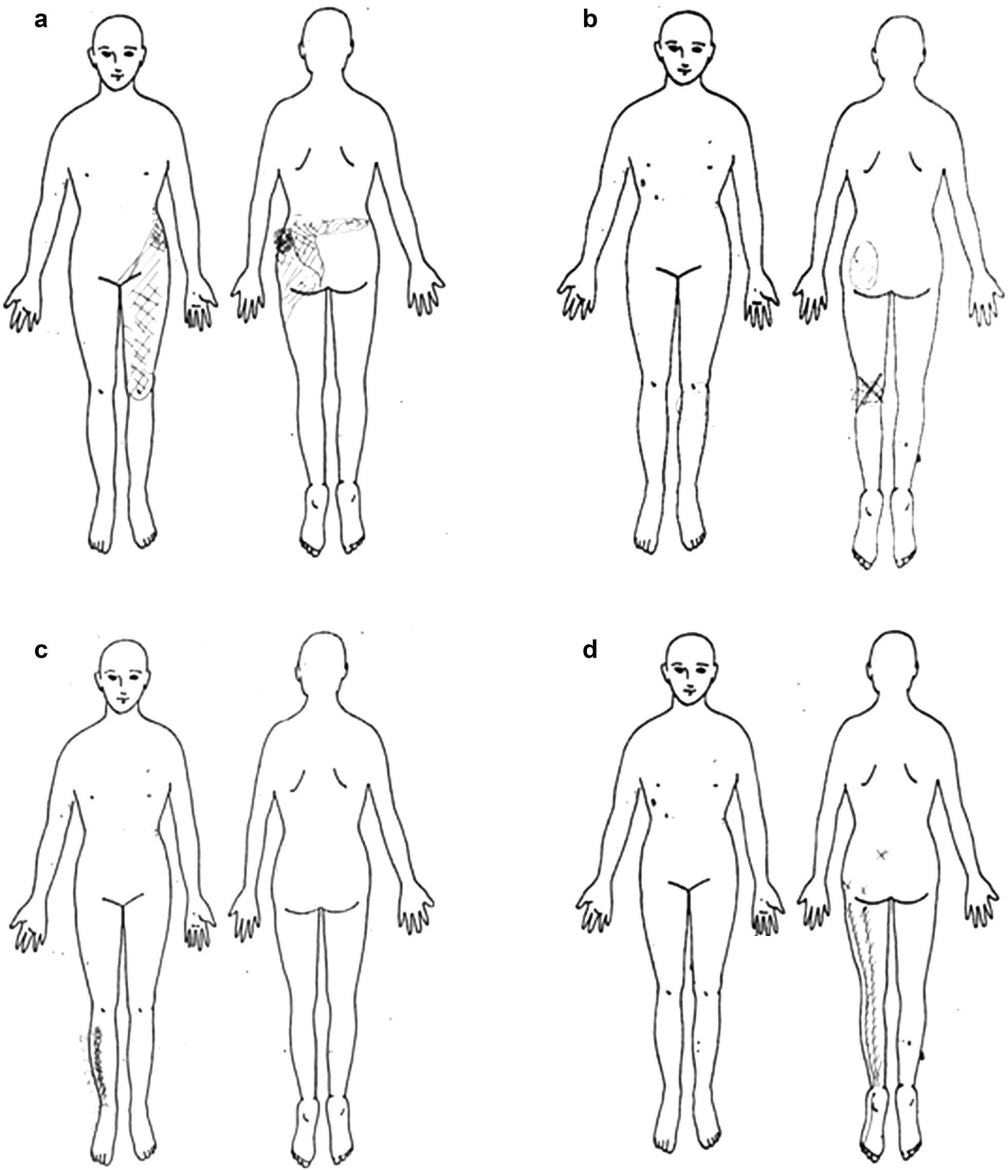
A representative sample of pain generated by patients with a single level lumbar disc herniation for each level. **a** = L2/3, **b** = L3/4, **c** = L4/5, **d** = L5/S

**Fig. 2:**
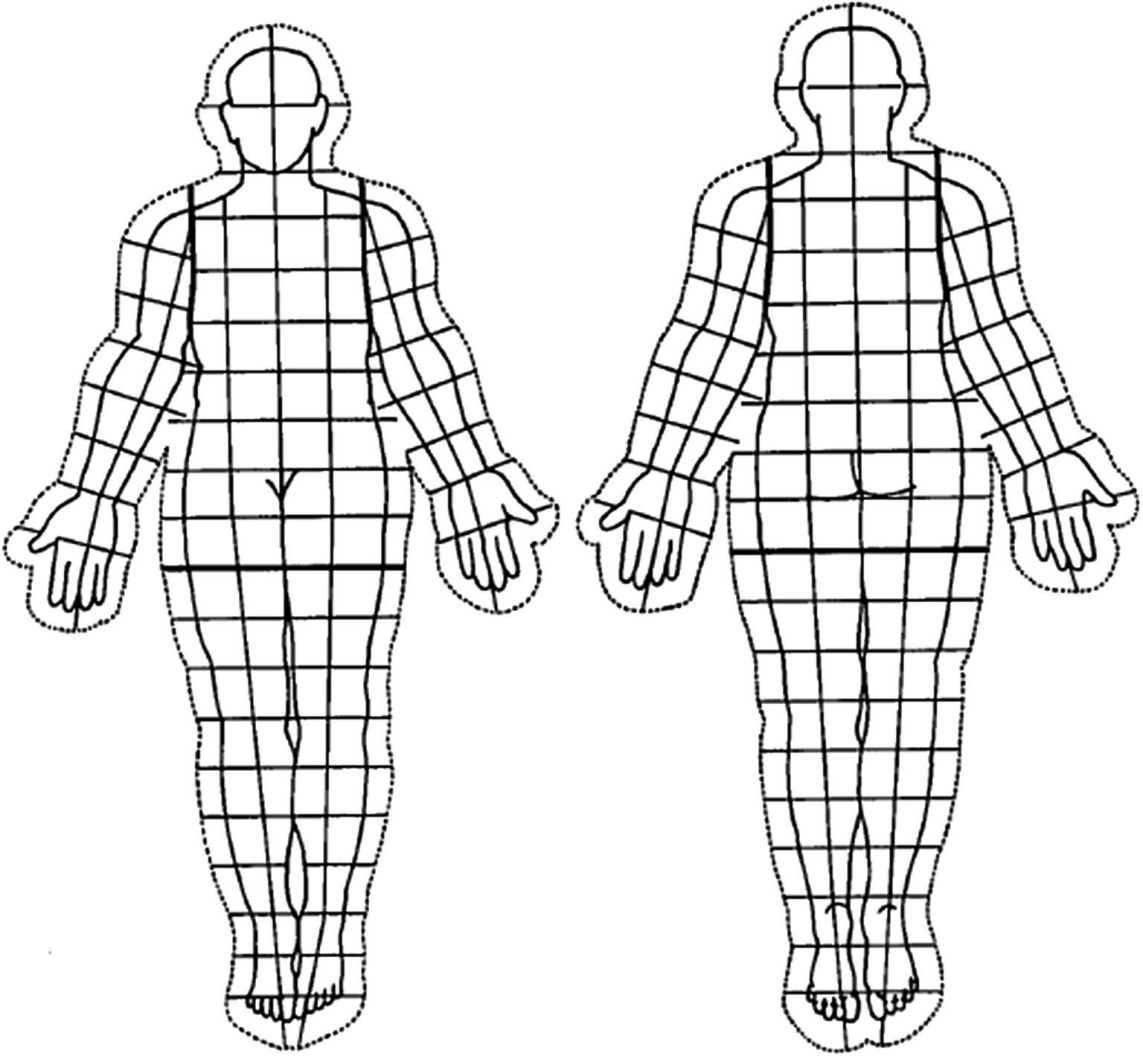
The grid score. The transparent overlay used to establish scores for patient’s pain drawing (Gatchel et al. 1986)

Among 54 consecutive patients with confirmed diagnosis of single level LDH who underwent treatment by the first author (TT) from 2006 to 2011, 31 patients who were treated conservatively by medication and epidural block were included in the study. Twenty-three patients who received surgery were excluded from the study. Medical records of these patients were evaluated retrospectively. PD evaluations were performed before and during the course of the treatment for each patient. Nurses explain to patients how to draw a PD at our outpatient clinic each time. Patients can draw on an area on the whole-body figure where they feel pain (Fig. 1). Three spine surgeons who were not informed of any other clinical information inferred the level of LDH by evaluating the PD results. They evaluated only the PDs which patients drew before treatment, and inferred the level of LDH of each patient. The first author determined the level of LDH based on the clinical information derived from neurological examination and MRI. Thereafter, correspondence between the two assessments (PD and clinical diagnosis) was examined. The levels of LDH among the included 31 patients were L2/3 in 4 cases, L3/4 in 3 cases, L4/5 in 8 cases, and L5/S in 16 cases (Fig. 1). Among the 31 patients included in the study, the clinical course during the treatment could be tracked for a minimum of 6 months for 26 patients, while the remaining 6 patients were lost to follow-up. The time period from the initial PD evaluation to the post-treatment evaluation ranged from 6 to 20 months. In order to quantitatively evaluate the severity of symptoms based on the PD results, GS was applied to the PD drawing (Fig. 2) (Gatchel et al. 1986). Boxes that are bilaterally symmetric and of approximately equal area cover the pain drawing, but also the quantitate pain extending outside the body, allows for differentiation of localized mechanical and referred/radicular pain patterns. The total numbers of boxes is 208. The numbers of boxes with drawings on the pain drawing was identified as GS. As comparative assessment measures, the Japanese Orthopaedic Association scoring system for low back pain (JOA score) with a maximum score of 29 (Hioki et al. 2011) and VAS were utilized. The maximum score of the JOA scoring system is 29 points (29 points means no physical symptoms), which is based on three subjective symptoms (9 points), three clinical signs (6 points), and seven activities of daily living (14 points) (Hioki et al. 2011). GS in PD and the JOA score as well as VAS before and after the treatment were comparatively analyzed. Moreover, the correlation between the GS, JOA score, and VAS was statistically assessed. This study was approved by our institutional review board, and informed consent was obtained from each patient. In the statistical analysis, the *t* test for improvements of GS, VAS and JOA score after treatments was performed with Excel (Microsoft Corporation, Redmond, WA, USA), and a regression analysis for correlation among GS, VAS and JOA score was performed using SPSS (SPSS, Chicago, IL, USA). A *p*-value of < 0.05 was considered to indicate significance.

## Results

**Fig. 3:**
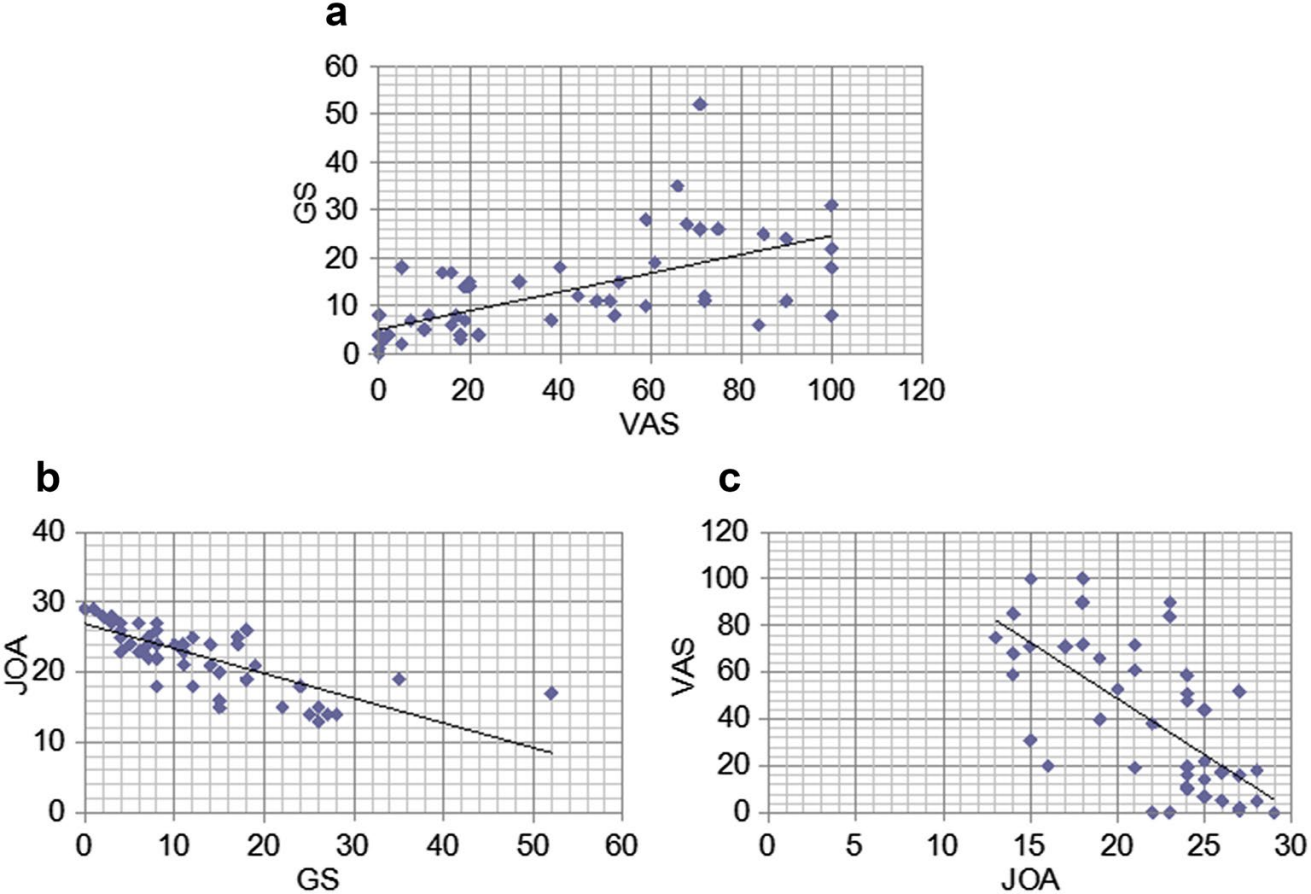
**a** GS and VAS have a significant positive correlation (r = 0.628, *P* < 0.05). **b** GS and the JOA score have a significant negative correlation (r = −0.764, *P* < 0.05). **c** VAS and the JOA score have significant negative correlation (r = −0.717, *P* < 0.05). *GS* indicates grid score. *VAS* indicates visual analogue scale. *JOA* score indicates the Japanese Orthopaedic Association scoring system for low back pain

The average age of 31 patients was 55 years olds (24–72 years olds). There were 21 males and 10 females. The accuracy of the PD assessment for the affected level calculated for each of the three raters ranged from 65.6 to 71.9 % with an average value of 68.8 ± 3.1 % (Table 1). The accuracy value for each LDH level was 33.3 % at L2/3, 33.3 % at L3/4, 90.5 % at L4/5, and 83.3 % at L5/S (Table 2). GS of all patients was evaluated from PD using a grid by the first author (Fig. 2). The average GS in all patients was 18.8 before treatments, and significantly decreased to 6.4 after treatment (*P* < 0.05). The average VAS significantly decreased after the treatment (64.2–13.5, *P* < 0.05). The JOA score also significantly improved after the treatment (18.4–26.3, *P* < 0.05). In assessment of the correlation among the different clinical assessment measures, significant positive correlations were demonstrated between GS and VAS both before and after the treatment (r = 0.628, *P* < 0.05, Fig. 3a). Moreover, a significant negative correlation was present between GS and the JOA score as well as VAS and the JOA score (r = −0.764, *P* < 0.05 Fig. 3b; r = −0.717, *P* < 0.05, Fig. 3c, respectively).

**Table 1:** Diagnostic accuracy of pain drawing for each rater

	Accuracy
Rater 1	23/32 (71.9 %)
Rater 2	21/32 (65.6 %)
Rater 3	22/32 (68.8 %)
Average	68.8 %

**Table 2:** Diagnostic accuracy of pain drawing in determining the affected level

Level	Accuracy
L2/3	4/12 (33.3 %)
L3/4	3/9 (33.3 %)
L4/5	19/21 (90.5 %)
L5/S	40/48 (83.3 %)

## Discussion

The significance of PD in the subjective evaluation of patients with low back disorders has been examined in a number of clinical studies. Those studies dealt with various morbidities such as benign low back pain, LDH, lumbar spinal stenosis, and psychological low back pain. Mann III et al. investigated the predictive value of PD in differentiating 5 disorder categories. They reported that the differential diagnosis by PD archived diagnostic accuracy of only about 50 % as compared with a physician’s assessment and computerized analytic methods (Mann et al. 1993). Rankine et al. investigated the diagnostic value of PD in assessment of nerve root compression as compared to MRI results. Consequently, the diagnostic accuracy of PD in the identification of affected nerve roots reached only 58 %, and these authors concluded that PD was not a good predictor of nerve compression as assessed by MRI results (Rankiene et al. 1998). Consequently, the reported accuracy of PD in assessment of patients with low back disorders has not been very high. However, these previous studies included patient populations of mixed morbidities, and the significance of PD assessment for each of the various low back disorders has not been well clarified. Therefore, in the present study, the study population was limited to patients with LDH involving a single nerve root. In the determination of the affected nerve root, the overall predictive value and diagnostic accuracy of PD were similar among the 3 raters. The overall diagnostic accuracy averaged 68.8 %, and this value was higher than that reported in previous studies dealing with mixed patient populations. Among the different levels, the accuracy as well as reliability for diagnosis was higher in L4/5 and L5/S than in L2/3 and L3/4. Regarding the innervation pattern for each nerve root, it has been shown that pain distribution arising from the L5 root and the S1 root is localized to the lateral aspect of the leg and the posterior aspect of the leg and the foot respectively (Schirmer et al. 2011; Vucetic et al. 1995). Taylor et al. investigated the pain distribution pattern revealed by PD for patients with unilateral leg pain due to LDH or lumbar spinal stenosis. They showed that 68 % of the patients with L4/5 lesion complained of pain in the anterior lower leg, while 75 % of the patients with L5/S lesion presented with posterior foot pain (Taylor et al. 1984). Judging from these study results, it is thought that pain arising from the L4/5 or L5/S level may be well discriminated by its distribution pattern. By contrast, the distribution pattern in patients with L2/3 or L3/4 involvement may be inconsistent resulting in lower diagnostic accuracy at these levels. Consequently, it may be concluded that PD assessment for the affected LDH level is reasonably accurate when the patient population is limited to those with LDH involving a single nerve root, especially at the L4/5 or L5/S level.

In the second part of the present study, the significance of GS in the quantitative assessment of PD was evaluated as compared to other clinical parameters such as the JOA score and VAS. All of these clinical evaluation measures showed significant improvement during the course of the conservative treatment. In the comparative analysis of these evaluation tools, GS was shown to correlate well with VAS and the JOA score. Previous studies have shown that the PD score can reflect the response to the treatment in patients with low back pain. Gatchel et al. demonstrated that trunk and leg PD scores in patients with low back pain who completed the therapeutic rehabilitation program significantly improved in accordance with other psychological and physical parameters (Gatchel et al. 1986). Ohnmeiss et al. showed that PD score assessed for the leg decreased after spinal cord stimulation in patients with chronic low back and leg pain (Ohnmeiss 2000). Thus, PD may be a useful tool for the evaluation of treatment outcomes during the treatment course in patients with sciatica.

The strengths of this study were the inclusion of a single surgeon’s series of uniform etiology and treatment. This study design helped improve consistency of data and quality of analysis. Furthermore, psychological factors can influence patients’ perception giving rise to inconsistency in the results; however, the patient population of the present study was limited to conservatively treated patients with single level LDH for a short follow-up period. Therefore, the effects of confounding variables on the results, such as psychological factors, could be minimized.

The limitations of the present study include the retrospective study design and the small sample size. In particular, small sample size might have affected the subgroup analysis of LDH levels. Moreover, another limitation is that the present study was based on the first author’s judgment of clinical sign and MRI findings. Additionally, 6 of the 31 included patients (19 %) were lost to follow-up. A prospective and large size study with a higher follow-up rate would be required for further validation of the usefulness of PD as a diagnostic tool for sciatica.

## Conclusions

The present study demonstrated the significance of PD in determining the affected level for patients with LDH involving a single nerve root, especially for LDH at L4/5 and L5/S. The results of the quantitative evaluation of PD using GS significantly correlated with the JOA score and VAS both before and after the conservative treatment, which indicates the potential usefulness of PD in clinical assessment during the treatment course.
